# Differential Rearrangement of Excitatory Inputs to the Medial Prefrontal Cortex in Chronic Pain Models

**DOI:** 10.3389/fncir.2021.791043

**Published:** 2021-12-24

**Authors:** Taylor Jefferson, Crystle J. Kelly, Marco Martina

**Affiliations:** ^1^Department of Neuroscience, Feinberg School of Medicine, Northwestern University, Chicago, IL, United States; ^2^Aptinyx Inc., Evanston, IL, United States; ^3^Department of Psychiatry and Behavioral Sciences, Feinberg School of Medicine, Northwestern University, Chicago, IL, United States

**Keywords:** thalamus, hippocampus, amygdala, mPFC, glutamate, prelimbic, infralimbic

## Abstract

Chronic pain patients suffer a disrupted quality of life not only from the experience of pain itself, but also from comorbid symptoms such as depression, anxiety, cognitive impairment, and sleep disturbances. The heterogeneity of these symptoms support the idea of a major involvement of the cerebral cortex in the chronic pain condition. Accordingly, abundant evidence shows that in chronic pain the activity of the medial prefrontal cortex (mPFC), a brain region that is critical for executive function and working memory, is severely impaired. Excitability of the mPFC depends on the integrated effects of intrinsic excitability and excitatory and inhibitory inputs. The main extracortical sources of excitatory input to the mPFC originate in the thalamus, hippocampus, and amygdala, which allow the mPFC to integrate multiple information streams necessary for cognitive control of pain including sensory information, context, and emotional salience. Recent techniques, such as optogenetic methods of circuit dissection, have made it possible to tease apart the contributions of individual circuit components. Here we review the synaptic properties of these main glutamatergic inputs to the rodent mPFC, how each is altered in animal models of chronic pain, and how these alterations contribute to pain-associated mPFC deactivation. By understanding the contributions of these individual circuit components, we strive to understand the broad spectrum of chronic pain and comorbid pathologies, how they are generated, and how they might be alleviated.

## Introduction

Chronic pain is one of the conditions with the highest negative impact on quality of life throughout the world. WHO data show that globally, lower back pain and migraines constitute the two leading causes of years lived with disability (GBD 2016 Disease and Injury Incidence and Prevalence Collaborators, 2017). Unfortunately, effective treatments for chronic pain are still lacking, due in large part to the incomplete understanding of the underlying pathogenic mechanisms. A fundamental difference between pain and other percepts is that there is no pain cortex; on the contrary, pain appears to be the perceptual creation originating from the complex information flow between multiple brain areas (Singh et al., 2020). This is not surprising, given that normal pain processing functions as a signal of danger. Healthy fear learning in response to a pain-inducing stimulus requires integration of sensory information (Where am I hurt?), information about the context (Where is the pain-inducing stimulus located?), and emotional salience (How much damage can this cause?). Therefore, in pain, and in chronic pain in particular, the sensory perception is inextricably mixed with cognitive symptoms and with a powerful emotional component (Berryman et al., 2013; Porreca and Navratilova, 2017; Bell et al., 2018). Consequently, the focus of pain researchers has widened to include brain areas beyond the ones strictly involved in somato-sensory perception. Among these new areas of interest, the ventral areas of the mPFC, the prelimbic (PL) and infralimbic (IL) sub-regions, are particularly intriguing. Due to the diverse functions of the major inputs to mPFC, these regions are ideally situated to process the multi-faceted information relevant to pain perception. Furthermore, the mPFC has been shown to mediate multiple components of chronic pain including sensory (Cordeiro Matos et al., 2015; Lee et al., 2015), cognitive (such as memory and attention problems, Moriarty et al., 2011; Baker et al., 2016) and emotional (such as catastrophizing, Galambos et al., 2019) components.

Current models suggest that functional deactivation of at least part of the ventral mPFC is a major pathogenic mechanism in different chronic pain conditions. A first hint of the major involvement of the PFC in chronic pain was provided about 15 years ago, when Apkarian et al. (2004) showed that the PFC of back pain patients shows a gray matter loss that is proportional to the duration of the pain. Subsequent animal studies confirmed the mPFC involvement and provided evidence suggesting a functional deactivation of the ventral areas of the mPFC early in the pain chronification process, although some differences exist between layer 2/3 and layer 5 neurons. In layer 2/3 pyramidal cells, a decreased α-amino-3-hydroxy-5-methyl-4-isoxazolepropionic acid (AMPA) and *N*-methyl-D-aspartate (NMDA) ratio, a measure of glutamatergic synaptic strength (Bredt and Nicoll, 2003; Kauer and Malenka, 2007), and decreased intrinsic excitability (Metz et al., 2009; Wang et al., 2015), together with an overall decrease in glutamate concentration in the PL (Kelly et al., 2016), suggest that both synaptic and intrinsic excitability are reduced. In layer 5 neurons, the decreased synaptic excitability (Kelly et al., 2016) and impaired excitatory cholinergic modulation (Radzicki et al., 2017) are only in part countered by a slight increase in intrinsic excitability (Cordeiro Matos et al., 2015; Wu et al., 2016). Mechanistic studies of chronic pain suggest that the ventral mPFC output exerts a critical modulatory role on pain perception, mostly through activation of descending pathways (Cheriyan and Sheets, 2018; Huang et al., 2019). This conclusion is supported by the fact that, in rodent pain models, optogenetic activation of the ventral mPFC has analgesic effect (Lee et al., 2015; Zhang et al., 2015). The cellular mechanisms of the region’s deactivation in chronic pain remain incompletely understood, but, as noted, abundant evidence suggests that synaptic mechanisms provide a major contribution to this functional state (Ji et al., 2010; Kelly and Martina, 2018). In this context, alteration of glutamatergic inputs to the mPFC appears to have a central role in the development of the chronic pain phenotype.

What are the major glutamatergic inputs to the ventral mPFC? The rodent PFC was originally defined as the cortical area receiving inputs from the medio-dorsal thalamus (Leonard, 1969; Krettek and Price, 1977). However, the thalamic inputs are not the only glutamatergic afferents to the ventral mPFC, and important projections originate in several other brain areas, including the contralateral mPFC, the amygdala, and the ventral hippocampus. Other inputs include the ipsilateral agranular insular cortex, which provides glutamatergic inputs to the PL (Hoover and Vertes, 2007), and the claustrum, which provides inputs to both the IL and PL. All four major inputs provide monosynaptic contacts onto dendritic spines on layer 2/3 pyramidal neurons (Little and Carter, 2012), as well as targeting neurons in deeper layers (Bacon et al., 1996; Parent et al., 2010; Collins et al., 2018; Kelly and Martina, 2018). Contacts with neurons whose cell bodies are located in deeper layers are the result of both direct innervation of these layers and of terminations onto the long apical dendrites of pyramidal cells that reach all the way to layer 1. For example, the widely branched apical tufts of layer 5 pyramidal neurons receive thalamic inputs up to layer 1 (Parent et al., 2010; Cruikshank et al., 2012).

Here we briefly summarize how the three main extra-cortical glutamatergic synaptic inputs (thalamic, hippocampal, and amygdalar) to the ventral mPFC are affected in rodent models of chronic pain and discuss how these changes may contribute to the chronic pain phenotype.

### Thalamic Inputs

**FIGURE 1:**
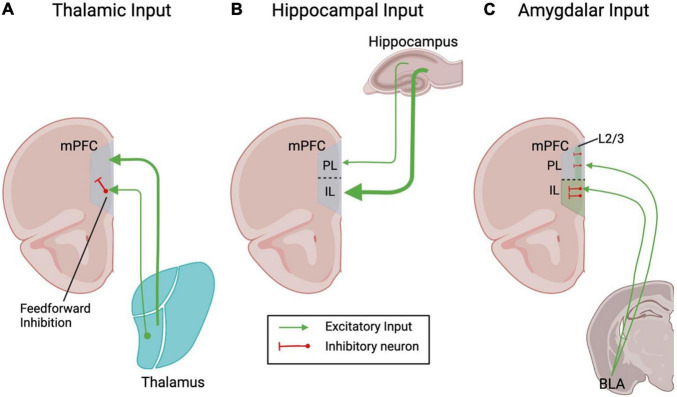
Schematic depicting the main extracortical glutamatergic inputs to the rodent mPFC. Line thickness represents the connection strength.

The major source of sensory input to the mPFC comes from the thalamus, which projects to both the PL and IL subregions of the mPFC (Krettek and Price, 1977). Retrograde tracer injections in the PL and IL result in heavy staining of multiple thalamic nuclei including the anteromedialis, mediodorsalis, nucleus rhomboideus and nucleus reuniens. The major thalamic afferents to the mPFC originate in the mediodorsal thalamus (MD) (Hoover and Vertes, 2007) and make contacts with dendritic spines, with the highest density in layer 3. Thalamic inputs target both pyramidal neurons and inhibitory interneurons (Figure 1A), so the net functional effect of their activation is mixed. For example, optogenetic activation of MD thalamic inputs causes both large glutamatergic and GABAergic currents in PL pyramidal cells (Kelly and Martina, 2018). The synaptic delay of the GABAergic currents suggest that they are at least in part mediated by feed-forward inhibition. The thalamus contacts parvalbumin (PV) expressing interneurons to evoke feedforward inhibition on mPFC (Canetta et al., 2020). Somatostatin (SST) and vasoactive intestinal peptide (VIP) expressing interneurons also receive direct glutamatergic inputs (Sun et al., 2019; Canetta et al., 2020). The timing and relative strengths of thalamic excitatory input and feed-forward inhibition mediate the thalamo-cortical rhythm that is critical for normal brain functioning. Accordingly, dampening thalamic activity causes significant reductions of GABA signaling in the mPFC and concomitant abnormalities in cognition and social interaction. These can be ameliorated by selectively activating mPFC PV-positive interneurons (Ferguson and Gao, 2018). Thalamic inputs to mPFC undergo both short-term and long-term bidirectional plasticity. For example, glutamatergic inputs from the MD and nucleus reuniens can undergo both long-term potentiation (LTP) and long-term depression (LTD) (Bueno-Junior et al., 2012; Vu et al., 2020). Glutamatergic inputs from the nucleus reuniens of the thalamus to layer 5 neurons show short-term depression in acute slice preparation when stimulated at theta frequencies (when using a 10 stimuli train at 5–10 Hz, the response to the last stimulus is roughly 60% of the first). These thalamic inputs cause very similar short term plasticity in layer 2/3 neurons (Banks et al., 2021). Thalamic inputs onto layer 5 pyramidal cells from the MD thalamus are also depressing, even at longer time intervals (Kelly and Martina, 2018).

At least in the early stage of neuropathic pain (1 week in rats) the overall magnitude of the excitatory thalamic glutamatergic input to PL pyramidal neurons appears reduced (Kelly and Martina, 2018; Table 1). Inhibition is also reduced, but whether this is driven by a reduction in feed-forward inhibition or by reduction in inputs to specific interneuronal populations remains unclear. In this context, it is worth noting that in a rodent model of acute pain, projections from the paraventricular thalamus to the mPFC appear to selectively enhance activation of GABAergic neurons suggesting thalamo-cortical feedforward inhibition in visceral nociception. Similar to the chronic pain scenario, pharmacogenetic activation of mPFC glutamatergic neurons attenuates visceral nociception (Jurik et al., 2015). In line with the hypothesis that the thalamic input to the mPFC is altered in chronic pain, a human brain imaging study (Henderson et al., 2013) found thalamic volume loss in patients with chronic neuropathic pain and suggested that chronic pain is associated with altered thalamic anatomy and impaired thalamo-cortical network activity.

**TABLE 1:** Main pain-associated alterations in mPFC glutamatergic inputs.

mPFC input	Inhibition-to-excitation ratio		Excitatory response	Inhibitory response	Short-term plasticity	Human imaging changes in pain
			
	Naïve		Changes in pain models			
Thalamic	2.3	No significant change	2-fold reduction	2-fold reduction	No significant change in probability of release	Reduced thalamic volume; reduced connectivity
	Model: Rat SNI, 1 week post-surgery (Kelly and Martina, 2018)					Trigeminal nerve pain patients (Henderson et al., 2013)
Hippocampal	0.6	No significant change	No significant change	No significant change	Reduced probability of release	Reduced connectivity
	Model: Rat SNI, 1 week post-surgery (Kelly and Martina, 2018)					Chronic back pain patients (Ayoub et al., 2019)
Amygdalar	1.2	2.1	No significant change	2-fold increase	Unknown	Increased connectivity
	Model: Rat arthritis model, 5–6 h post-kaolin and carrageenan injection (Ji et al., 2010)					Complex regional pain syndrome patients (Simons et al., 2014)

### Hippocampal Inputs

The hippocampal input to the mPFC provides an important anatomical substrate for learning and memory functions, as the hippocampus is required for memory formation and encoding, particularly of declarative memories. The mPFC is important for working memory function, and may link the hippocampal memory trace with other regions of the neocortex for long-term memory storage (Thierry et al., 2000). While there is no known direct mPFC projection to the hippocampus, the ventral region of the hippocampus provides an important glutamatergic input to the mPFC (Parent et al., 2010). The hippocampal afferents to the mPFC contact both pyramidal cells and interneurons in layers 2–6 (Parent et al., 2010; Kelly and Martina, 2018; Canetta et al., 2020) and have a distinct spatial pattern, with the densest innervation in the ventral region of mPFC and gradually more sparse innervation more dorsally (Jay and Witter, 1991; Figure 1B). Thus, the excitatory effects of hippocampal inputs increase along the dorso-ventral axis so that in anesthetized rats stimulation of CA1 evokes an excitatory response in only 14% of cells in dorsal PL, which increases to 42% in the ventral part of PL and reaches 61% in IL (Ishikawa and Nakamura, 2003). The innervation of dorsal PL is overall sparser and by and large limited to layers 5–6 (Thierry et al., 2000, but see Canetta et al., 2020), while in the IL the afferents contact cells in layer 2/3 as well as layer 5–6. mPFC inputs have been explored in four separate pyramidal neuron populations delineated by their output targets: those that project to contralateral cortex (cortico-cortical), those that project to the amygdala (cortico-amygdalar), those that project to striatum (cortico-striatal), and those that project to the pons (cortico-pontine). In layer 2/3 of IL, the hippocampal inputs contact both cortico-cortical and cortico-amygdalar neurons (Liu and Carter, 2018). Interestingly, at least in PL layer 2/3, all cells receiving hippocampal inputs also receives MD thalamic inputs (Canetta et al., 2020). In layer 5 of both IL and PL the hippocampal inputs show a preference for cortico-cortical neurons (Liu and Carter, 2018). Both the subiculum and CA1 project to the PL region of the mPFC (Ferino et al., 1987), and CA1 also projects to the IL (Swanson, 1981). Additionally, the mPFC receives collaterals from the projection connecting the CA1/subiculum to agranular insular area of the lateral prefrontal cortex (Verwer et al., 1997). Overall, it has been estimated that the hippocampal inputs synapse onto approximately 40% of all mPFC neurons, including pyramidal neurons and interneurons. Consequently, a single stimulation in the hippocampus induces an early excitatory postsynaptic potential (EPSP) in pyramidal cells (Degenetais et al., 2003) as well as short latency excitatory responses in identified interneurons (Ferino et al., 1987; Tierney et al., 2004). Accordingly, when PL responses to hippocampal stimulation are recorded in anesthetized rats, PL pyramidal neurons respond with a single action potential that in ∼40% of cells is followed by prolonged inhibition. The inhibitory response appears the result of both feed-forward and feedback components. These responses exhibit paired-pulse facilitation and can undergo long-term potentiation in response to high frequency tetanic stimulation (Laroche et al., 1990). Interestingly, and in contrast with the thalamic inputs to the PL, which show a preference for PV-positive interneurons, the hippocampal inputs to the PL are skewed toward VIP-positive interneurons (Canetta et al., 2020).

Alterations in hippocampal–mPFC connections are reported in both human chronic pain patients (Mutso et al., 2014; Ayoub et al., 2019) and animal pain models (Cardoso-Cruz et al., 2013; Ma et al., 2019). Multielectrode array recordings in a rodent model of inflammatory pain shows that information flow from the ventral CA1 to the infralimbic cortex is reduced between 6 and 12 days after pain onset (Ma et al., 2019), in line with a previous finding suggesting that the information flow between the hippocampus and the mPFC is impaired in neuropathic pain rats (Cardoso-Cruz et al., 2013). Similarly, in chronic back pain patients, functional connectivity between the anterior hippocampus and mPFC is reduced (Ayoub et al., 2019). Hippocampal modulation of IL activity is disrupted in rats with peripheral inflammation, and chemogenetic activation of the glutamatergic hippocampal input to the mPFC reduces spontaneous pain (Ma et al., 2019). This finding suggests that in pain conditions the efficacy of hippocampal input to the mPFC is decreased, in line with the interpretation that the probability of release at this synapse is decreased in neuropathic pain (Kelly and Martina, 2018; Table 1).

### Amygdalar Inputs

The third major extracortical glutamatergic input to the mPFC comes from the amygdala, which is critical for processing fear and other negative emotions. The mPFC receives inputs from the medial portions of the basolateral nucleus (BLA), and adjacent portions of the lateral, basomedial and amygdalo-hippocampal nuclei (McDonald, 1991); the main amygdalar input to the ventral mPFC, however, originates in the BLA (Hoover and Vertes, 2007). Studies combining retrograde tracer injections into rat mPFC with immunohistochemistry for glutamate and aspartate found that the BLA sends glutamatergic projections to multiple mPFC subregions, including the anterior cingulate cortex (ACC), PL and IL (McDonald et al., 1996; Figure 1C). BLA inputs preferentially target layer 2 cortico-amygdalar over neighboring cortico-striatal neurons. Importantly, these afferents make even stronger connections onto neighboring PV- and SST-expressing interneurons, which in turn preferentially target cortico-amygdalar neurons (McGarry and Carter, 2016). In keeping with these findings, BLA glutamatergic projections to layers 2–6 of the rat mPFC establish synaptic contacts with dendritic spines of pyramidal neurons as well as with the aspiny dendritic shafts and somata of PV-positive neurons (Gabbott et al., 2006). Beside PV-interneurons, amygdalar inputs form monosynaptic contacts also on SST-positive interneurons of the ventral mPFC (McGarry and Carter, 2016). Accordingly, BLA inputs evoke excitatory and inhibitory responses in layer 5 pyramidal neurons of both the IL and PL (Cheriyan et al., 2016). BLA inputs show some degree of regional and laminar segregation, as they mostly target layer 2/3 in the PL (with limited presence in layer 5), but show generalized distribution in layer 5 of the IL. Thus, multiple lines of evidence show that BLA inputs to the PL and IL form monosynaptic contacts with both pyramidal neurons and interneurons, which results in a net inhibitory (Ji et al., 2010) metabotropic glutamate receptor 1 (mGluR1) dependent (Sun and Neugebauer, 2011) effect *in vivo*. These findings are further supported by *in vivo* recordings from the IL and PL of anesthetized rats, which show that pyramidal neurons respond to BLA stimulation with a combination of an early excitatory component and one or more inhibitory components. Interestingly, the excitatory component does not evoke action potentials, while the inhibition is long-lasting (∼300 ms), confirming that the net effect of the BLA stimulation is inhibitory (Dilgen et al., 2013).

Rodent studies have established a key role for enhanced BLA input in the pain-associated PFC deactivation. The first data were provided by an elegant study from the Neugebauer lab in a rat model of inflammatory pain. These authors demonstrated that, in this model, increased BLA activity leads to selective enhancement of inhibitory synaptic currents in PFC pyramidal neurons, without affecting the excitatory input (Ji et al., 2010). This effect may appear counterintuitive, as increased glutamatergic input leads to cortical deactivation, but it is in line with the net inhibitory effect of the BLA inputs to the mPFC and further supported by data obtained in a mouse model of neuropathic pain showing that optogenetic inhibition of synaptic inputs from the BLA to GABAergic interneurons in the mPFC have analgesic effect (Huang et al., 2019). Additionally, a very recent paper found that the ratio of excitatory to inhibitory effects elicited by activation of BLA inputs to the PL, but not the IL, is decreased in a rodent neuropathic pain model (Cheriyan and Sheets, 2020). In line with this scenario, synaptic input from the BLA to inhibitory interneurons in the PL increases in neuropathic pain due to reduced endocannabinoid-regulated mGluR5 modulation (Kiritoshi et al., 2016; Huang et al., 2019). Thus, it is likely that the increased functional connectivity between the left amygdala and multiple cortical regions, including the prefrontal and cingulate cortices demonstrated in a human neuroimaging study of chronic pain patients results in a net inhibitory effect (Simons et al., 2014).

## Discussion

Numerous findings support the idea that the ventral mPFC is deactivated in chronic pain conditions (Ji et al., 2010; Wang et al., 2015; Kelly et al., 2016; Radzicki et al., 2017). It is reasonable to hypothesize that the decreased function of the mPFC network is mediated by the compounded action of three distinct mechanisms. The first two mechanisms that contribute to the mPFC deactivation in the chronic pain condition are synaptic: a combination of input-specific depression of the excitatory glutamatergic inputs, and potentiation of GABAergic inhibition. The reduced glutamatergic drive might explain the shortened dendrites of layer 5 mPFC neurons (Kelly et al., 2016), which, possibly in combination with the reduced length of the axon initial segments (Shiers et al., 2018), may provide a cellular basis for the grey matter reduction associated with chronic pain in patients and animal models (Apkarian et al., 2004; Seminowicz et al., 2009).

The altered synaptic inputs may also cause a more general effect, which is a widespread alteration of the excitation/inhibition (E/I) balance. E/I imbalance is regarded as a key pathogenic mechanism in numerous neurodevelopmental disorders (Deidda et al., 2015; Kim et al., 2021). Thus, it is possible that through recurrent inhibitory and excitatory circuitries the mPFC network acts as a magnifier of the effects of even minor alterations in specific inputs, so that relatively minor synaptic changes end up producing large behavioral effects.

Clearly, these synaptic effects do not take place in a vacuum but interact with the neuronal intrinsic excitability. For example, many of the thalamic input to the ACC and the PL are provided by the same thalamic neurons, which form terminals in these two different cortical areas (Kuramoto et al., 2017); yet, in chronic pain conditions the ACC is hyperexcited (Zhao et al., 2006; Xu et al., 2008; Blom et al., 2014; Singh et al., 2020) and the PL is inhibited. This finding suggests that a third mechanism, which is represented by alterations in intrinsic excitability such as increased input resistance in the ACC (Santello and Nevian, 2015) or increased action potential threshold in the PL (Wang et al., 2015), contributes to the final electrophysiological outcome of altered synaptic inputs.

As to the functional consequences of the reduced mPFC output, it is likely that the most relevant effects on the sensory components of pain perception are mediated by the descending projections to the periaqueductal gray (PAG) and to the nucleus accumbens (NAc). This idea is supported by the finding that the inhibition of the mPFC output appears particularly strong in neurons projecting to the PAG (Cheriyan and Sheets, 2018). However, activation of the mPFC projection to the nucleus accumbens has analgesic effect by itself (Lee et al., 2015), as does direct optogenetic activation of the nucleus accumbens core (Kc et al., 2020); additionally, inhibition of the PFC input to the nucleus accumbens amplifies both sensory and affective symptoms of acute pain in naïve animals (Zhou et al., 2018). Thus, the exact identity of the brain networks that relay the mPFC deactivation-mediated modulation of sensory pain remains to be established.

The cognitive and affective components of the chronic pain syndrome are likely mediated by different mPFC projections, and the correlation between mPFC firing and cognitive performance appears different in control and pain conditions. For example, disruption of the ventral hippocampus to PFC connectivity was reported in a model of inflammatory pain (Ma et al., 2019). Additionally, theta-rhythm connectivity between the (dorsal) hippocampus and the mPFC is reduced in neuropathic pain, and this is correlated with performance on a working memory task (Cardoso-Cruz et al., 2013). As expected, in control conditions, optogenetic inhibition of PL firing and of hippocampus-mPFC coherence disrupts working memory performance. In pain animals, however, optogenetic inhibition of PL pyramidal cells restores normal theta rhythms and coherence and improves working memory (Cardoso-Cruz et al., 2019a). As the chronic pain phenotype (both neuropathic and inflammatory) is characterized by reduced mPFC excitability and disrupted ventral hippocampus–PL connectivity, the fact that pyramidal neuron inhibition improves cognitive performance in pain animals is unexpected and suggests a context where synaptic modulation of pyramidal cell activity, possibly through E/I imbalance (discussed above) is more important than simple firing frequency. Although the detailed network effects of this optogenetic modulation on PL output remain to be clarified, it is interesting that this manipulation does not directly affect pain measures (Cardoso-Cruz et al., 2019b). Another group, however, recently showed that modulation of the input from the ventral hippocampus to the mPFC has a significant impact on pain perception, as chemogenetic activation of the ventral CA1-IL pathway alleviated spontaneous pain in a model of inflammatory pain (Ma et al., 2019). Thus, these data suggest that input-specific effects are central to the mPFC role in pain modulation. At the same time, the role of the ventral hippocampus in pain perception also remains unclear, because in a rodent model of early stage neuropathic pain neither optogenetic nor pharmacological activation of the ventral hippocampus produced analgesic effects (Wei et al., 2021). Multiple factors may explain these differences, including the specific pain model used, the pain duration, the strength, duration and pattern of the hippocampal stimulation, and the type of neurons stimulated.

Thus, as expected for a complex percept such as pain, the brain mechanisms involved are extremely complicated. An additional layer of complexity is provided by the fact that although both the PL and IL are inactivated in chronic pain, it is unclear whether their relative function level is equally affected. For example, a recent study in a neuropathic pain model found that the balance between excitatory and inhibitory effects caused by activation of the BLA input to the mPFC is decreased in the PL, but not the IL (Cheriyan and Sheets, 2020). This is not a trivial point because the PL and IL have different functions. For example, in coding aversion responses, they are believed to play opposite roles, with the PL promoting aversion learning and the IL facilitating extinction of aversive responses (Gourley and Taylor, 2016). Whether similar differences exist in the roles that the IL and PL play in the pain phenotype remains unclear. The picture, however, is even more complex, as we must also consider the temporal structure of mPFC rearrangement in the pain phenotype. Many of the changes we have discussed take place relatively early in the course of the disease (in rodents, within 1–2 weeks from the onset of the peripheral injury). This is a particularly interesting timeframe, because it likely represents the transition from acute to chronic pain. Yet, brain networks continue to change in the presence of neuropathic injuries, either continuing along their disease trajectory or due to adaptive responses (Ren et al., 2021). Finally, the pain phenotype also exhibits important sex dimorphism, including in mPFC-specific tasks (Shiers et al., 2018). Accordingly, recent data show that sex-specific differences are apparent in pain-associated effects on GABAergic modulation in the rodent Mpfc (Jones and Sheets, 2020). Whether these sex-specific differences in mPFC function are relevant for the sex dimorphism of the chronic pain phenotype will likely represent an intriguing field of research in the near future.

## Author Contributions

CK focused on researching hippocampal inputs. TJ focused on amygdalar inputs. MM focused on thalamic inputs and drafted the manuscript. All authors contributed to the final version.

## Conflict of Interest

CK is employed by company Aptinyx Inc. The remaining authors declare that the research was conducted in the absence of any commercial or financial relationships that could be construed as a potential conflict of interest.

## Publisher’s Note

All claims expressed in this article are solely those of the authors and do not necessarily represent those of their affiliated organizations, or those of the publisher, the editors and the reviewers. Any product that may be evaluated in this article, or claim that may be made by its manufacturer, is not guaranteed or endorsed by the publisher.
